# Supplementary figures and images for: maxATAC: Genome-scale transcription-factor binding prediction from ATAC-seq with deep neural networks
Source: PLoS Comput Biol. 2023 Jan 31;19(1):e1010863. doi: 10.1371/journal.pcbi.1010863 (PMC9917285; doi:10.1371/journal.pcbi.1010863)

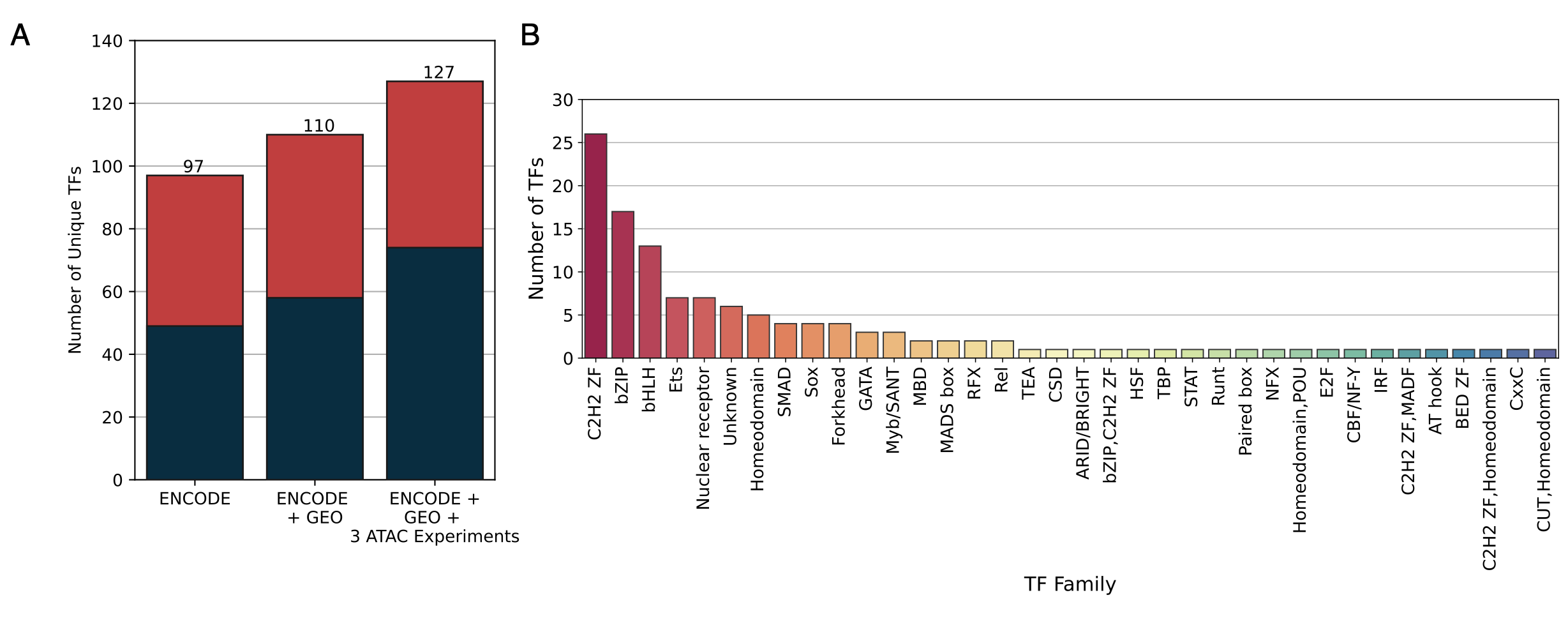

Supplement: S1 Fig — (A) The number of unique TF models (y-axis) that can be trained using different combinations of ENCODE, GEO, and in-house generated OMNI ATAC-seq data (x-axis). TFs are broken into two categories: (1) those with only 2 cell lines available (only cross-cell type training is feasible, red bar) and (2) models that have ≥ 3 cell lines (cross-cell type training and performance evaluation in a held-out test cell type are feasible, black bar). (B) The distribution of maxATAC TF models across TF families. The "unknown" category contains TFs that have not been associated with a TF family. (TIF) [file pcbi.1010863.s006.tif]

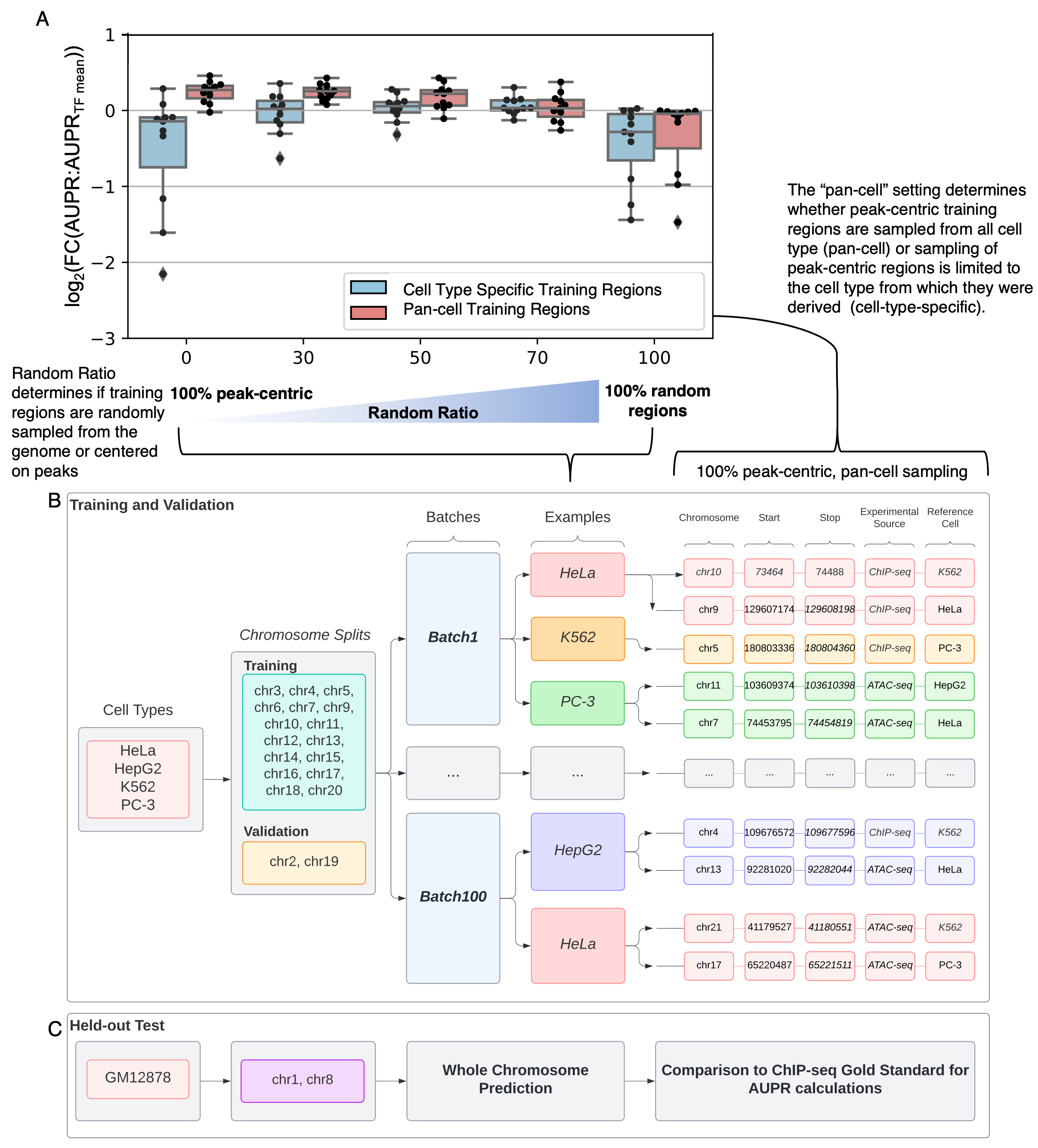

Supplement: S2 Fig — (A) A small subset of our benchmark (11 TFs) was used to test peak-centric, pan-cell training (Methods) in a held-out cell type, GM12878. We varied the relative ratio of random and peak-centric regions ("Random Ratio") sampled during model training. Random ratio of 100% indicates that all training examples were randomly sampled from the genome, while 0% indicates that only peak-centric regions (centered on ChIP-seq and ATAC-seq peaks in the training cell type) were used. "Peak-centric" training enriches for TP examples and potentially challenging TN (high ATAC-seq signal but no TFBS). Furthermore,”pan-cell” training (red) additionally enriches for challenging TN examples, by pooling peak-centric regions across the training cell type. During pan-cell training, peak-centric regions are randomly sampled using signal from any of the training cell types (which may or may not have a TFBS in a given peak-centric region). This is in contrast to "cell type-specific" training (blue), in which peak-centric regions are specific to each of the training cell types and sampling of a peak-centric region is limited to signal from the cell types from which the peak-centric region was originally identified. (B) shows an example of 100% peak-centric, pan-cell training. (Note how peak-centric examples from HeLa come from both K562 and HeLa.) (C) The held-out test cell type, in addition to chr1 and chr8, are independent of model training and thereby enable performance evaluation of maxATAC model predictions in new cell types. (TIF) [file pcbi.1010863.s007.tif]

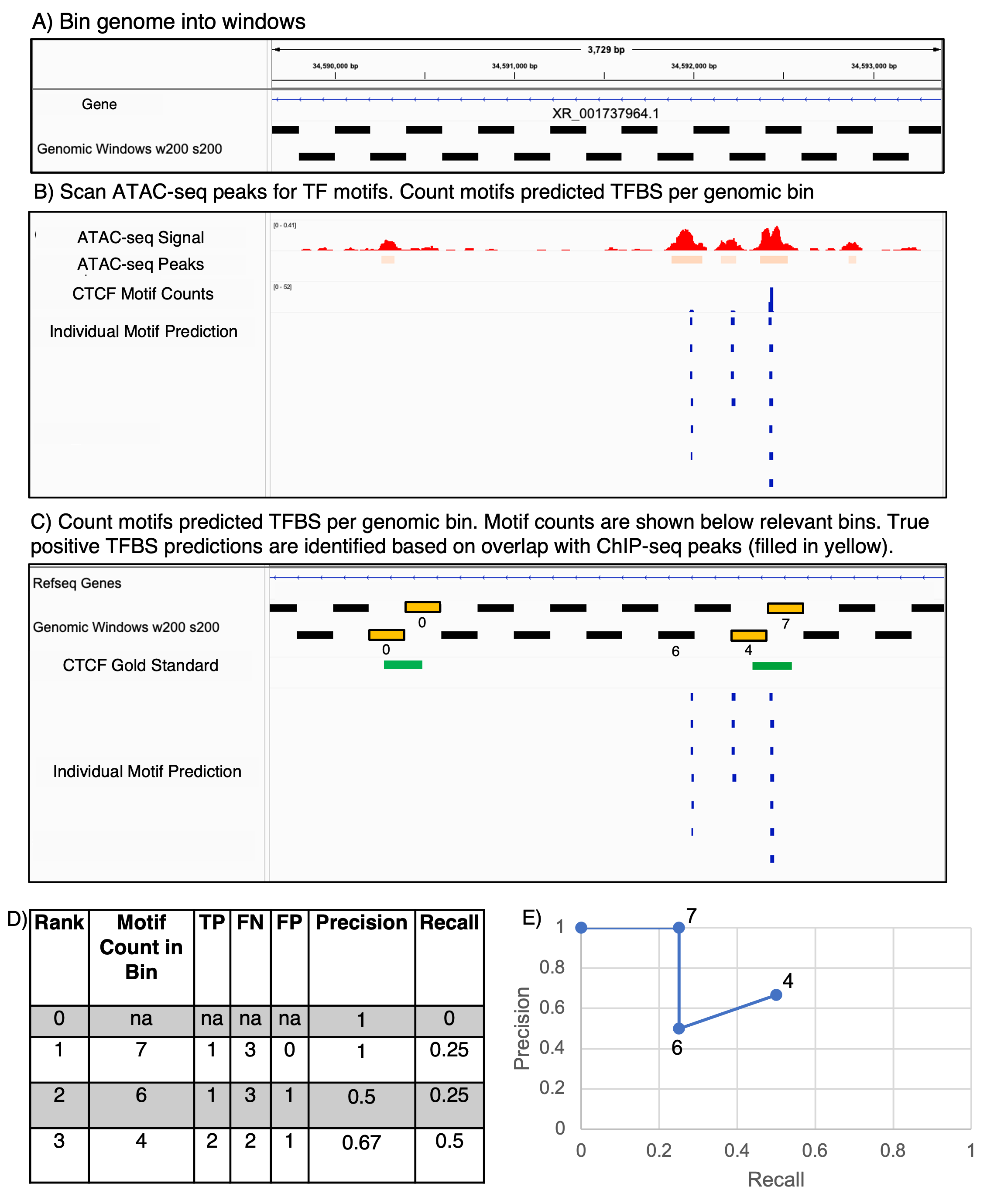

Supplement: S3 Fig — This figure shows how a precision-recall curve is constructed from motif predictions and uses artificial data for a small genomic window. (A) For this example, the genome is divided into bins of length 200bp and sliding windows of length 200bp; windows overlapping blacklisted regions are removed. (B) ATAC-seq peaks are scanned with MOODS using the CisBP version 2 database of TF motifs. (C) TFBS predictions are ranked based on the number of motif occurrences within the bin. For TFs with >1 motif available, overlapping motif occurrences are counted as a single occurrence. True positive TFBS predictions are identified as those overlapping ChIP-seq peaks (yellow fill). (D) Bins are ranked by the number of TFBS prediction and compared to the gold standard. (E) Precision and recall are calculated at each threshold and summarized as a precision-recall curve. (TIF) [file pcbi.1010863.s008.tif]

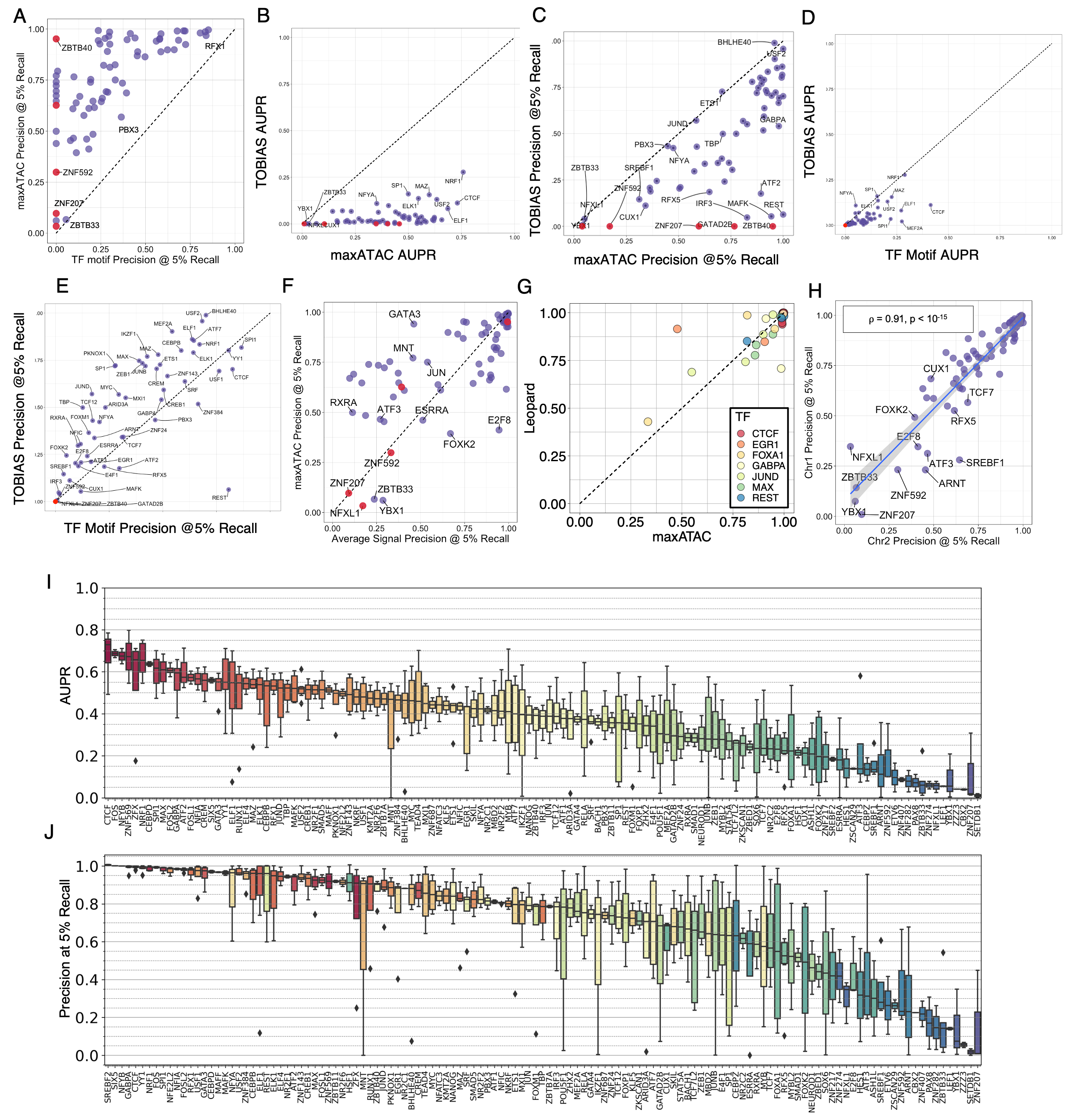

Supplement: S4 Fig — (A) Comparison of median test precision at 5% recall between maxATAC TFBS predictions and TF motif scanning, where median is the median performance across each possible train-test cell type split. (B-E) Performance comparison of TOBIAS to maxATAC or simple TF motif-scanning TFBS prediction. Chromosome-wide test performance (chr1) using OMNI-ATAC of GM12878 (50k cells) [28] is reported for 60 TFs. TFs that do not have a known motif are highlighted in red. Test (B) AUPR or (C) precision at 5% recall of TOBIAS versus maxATAC. Test (D) AUPR or (E) precision at 5% recall of TOBIAS versus TF motif scanning in ATAC-seq peaks. (F) Comparison of median test precision at 5% recall between maxATAC TFBS predictions and TFBS prediction using averaged training ChIP-seq signal. (G) Test performance (precision at 5% recall) of maxATAC models compared to Leopard (DNase-seq-based) models using ATAC-seq input and maxATAC ChIP-seq gold standards for 8 cell lines and 7 TFs. maxATAC outperforms Leopard for 17 out of 29 test performance comparisons. (H) Comparison of median test versus validation precision at 5% recall for maxATAC models constructed using all train-test cell type splits. Test corresponds to the test cell type and chromosome (chr1), while validation corresponds to training cell types and chromosome (chr2); ρ = 0.91 and P < 10−15, n = 74 TFs. Validation (I) AUPR (median = 0.43) and (J) precision at 5% recall (median = 0.85) for the final 127 maxATAC TF models. (TIF) [file pcbi.1010863.s009.tif]

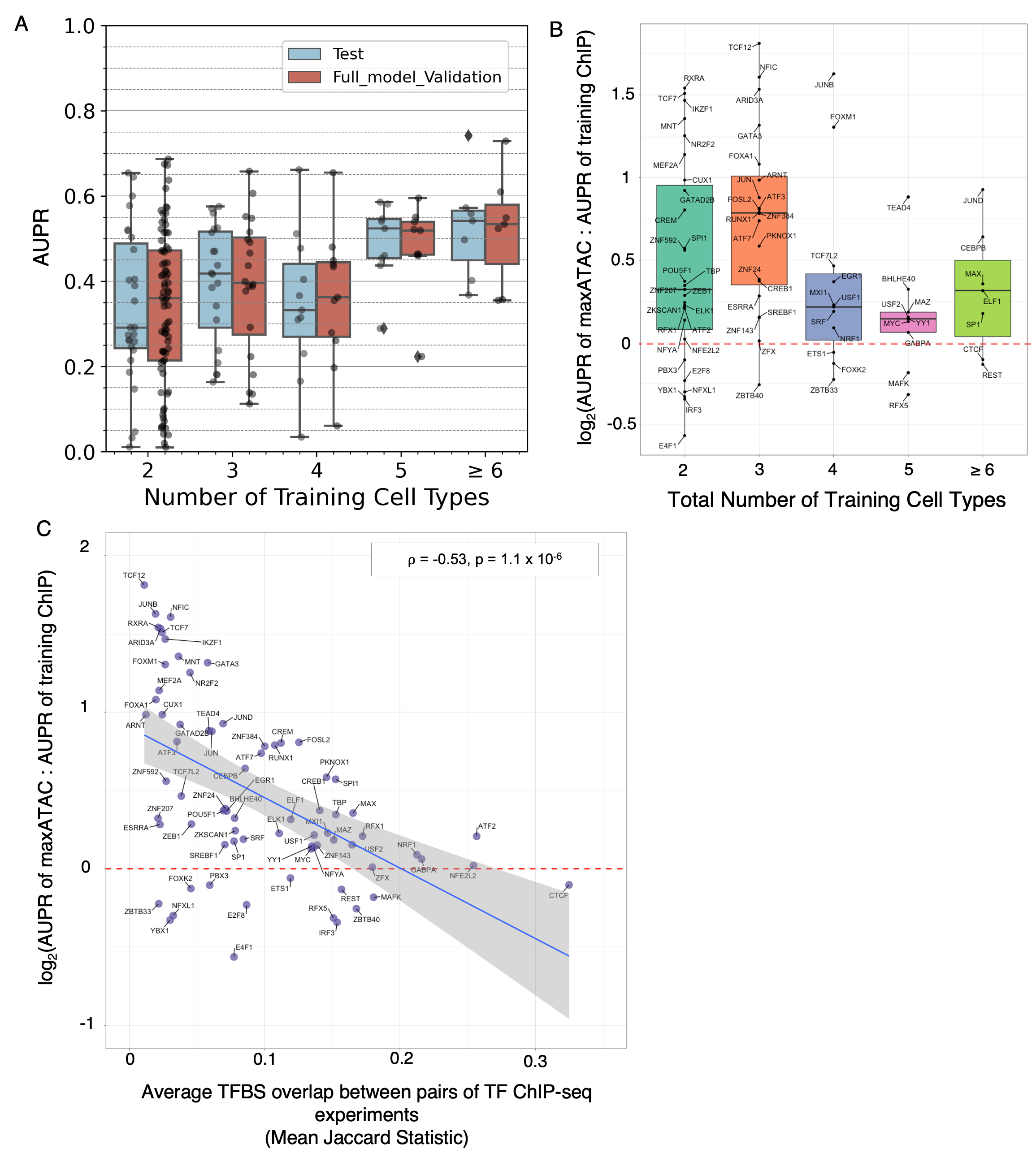

Supplement: S5 Fig — (A) AUPRMEDIAN of maxATAC models as a function of number of cell types available for training. Validation performance (red) is estimated for the final maxATAC models (using all cell types available for model construction), n = 127 TFs, while test performance (blue) is available for 74 TF. (B-C) Factors contributing to relative test performance differences between maxATAC and average training ChIP-seq signal (74 TFs, analysis of median test AUPR). (B) Log2-ratio of maxATAC AUPR relative to the AUPR of TFBS prediction using the averaged training ChIP-seq signal as a function of training cell types. (C) Log2-fold-change, of maxATAC AUPR relative to the AUPR of TFBS prediction using training ChIP-seq signal, versus averaged Jaccard overlap between TFBS (from ChIP-seq) in pairs of training cell types. (TIF) [file pcbi.1010863.s010.tif]

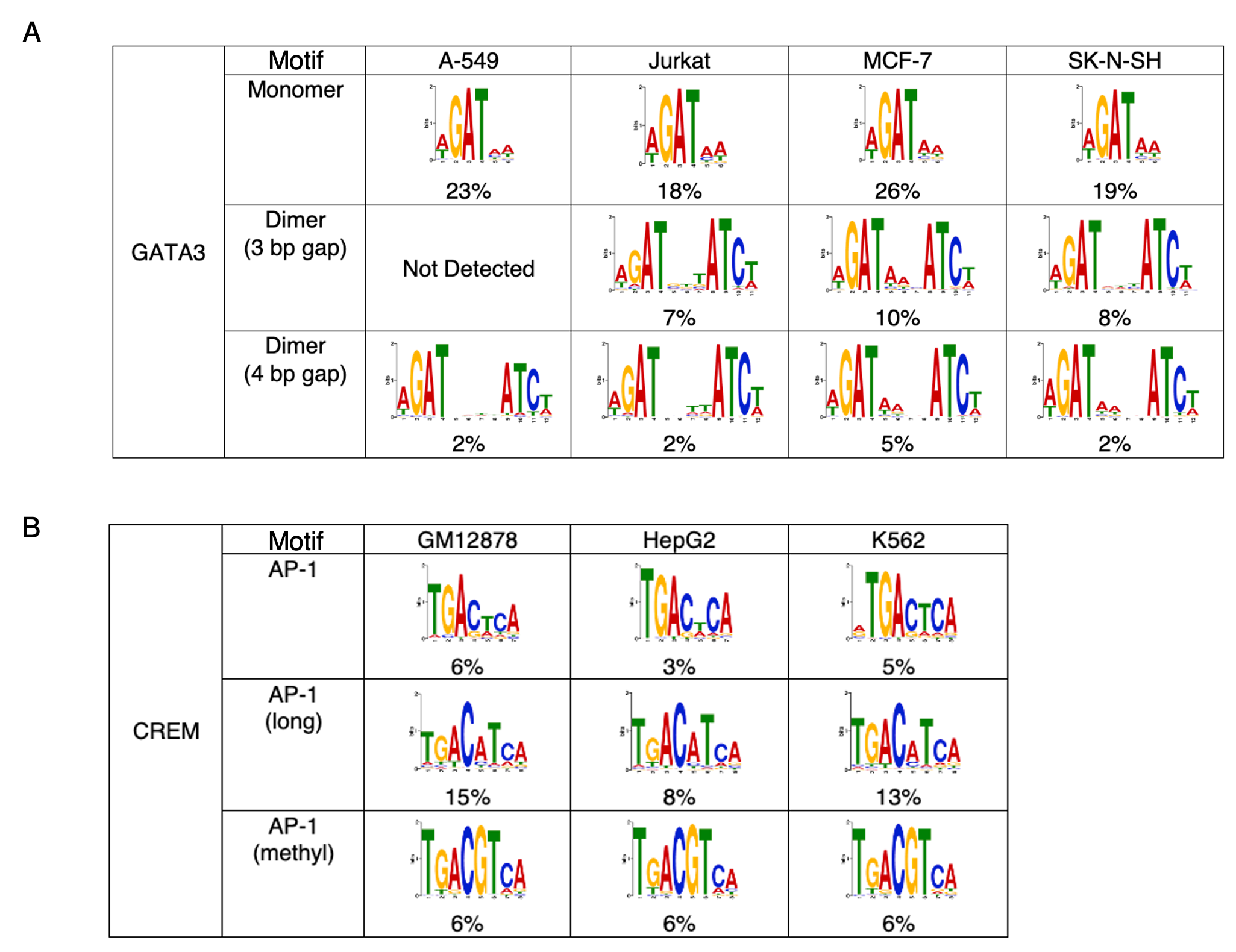

Supplement: S6 Fig — For (A) GATA3 and (B) CREM maxATAC models, we modified TF-MoDISco to uncover importance-weighted DNA sequence patterns (summarized as CWM logos). TF-MoDISco was run independently on each of the training cell types, using positive TFBS examples (ChIP-centered inputs) on test chromosomes 1 and 8. ATAC signals for those regions were held constant and CWM were derived using the 15th bin of our 32-dimensional, 32bp-resolved TFBS prediction output, which corresponded to the center of the TF ChIP-seq peak. Percentages below each CWM indicate the fraction of positive examples containing the CWM in the 15th bin for each cell type. (TIF) [file pcbi.1010863.s011.tif]

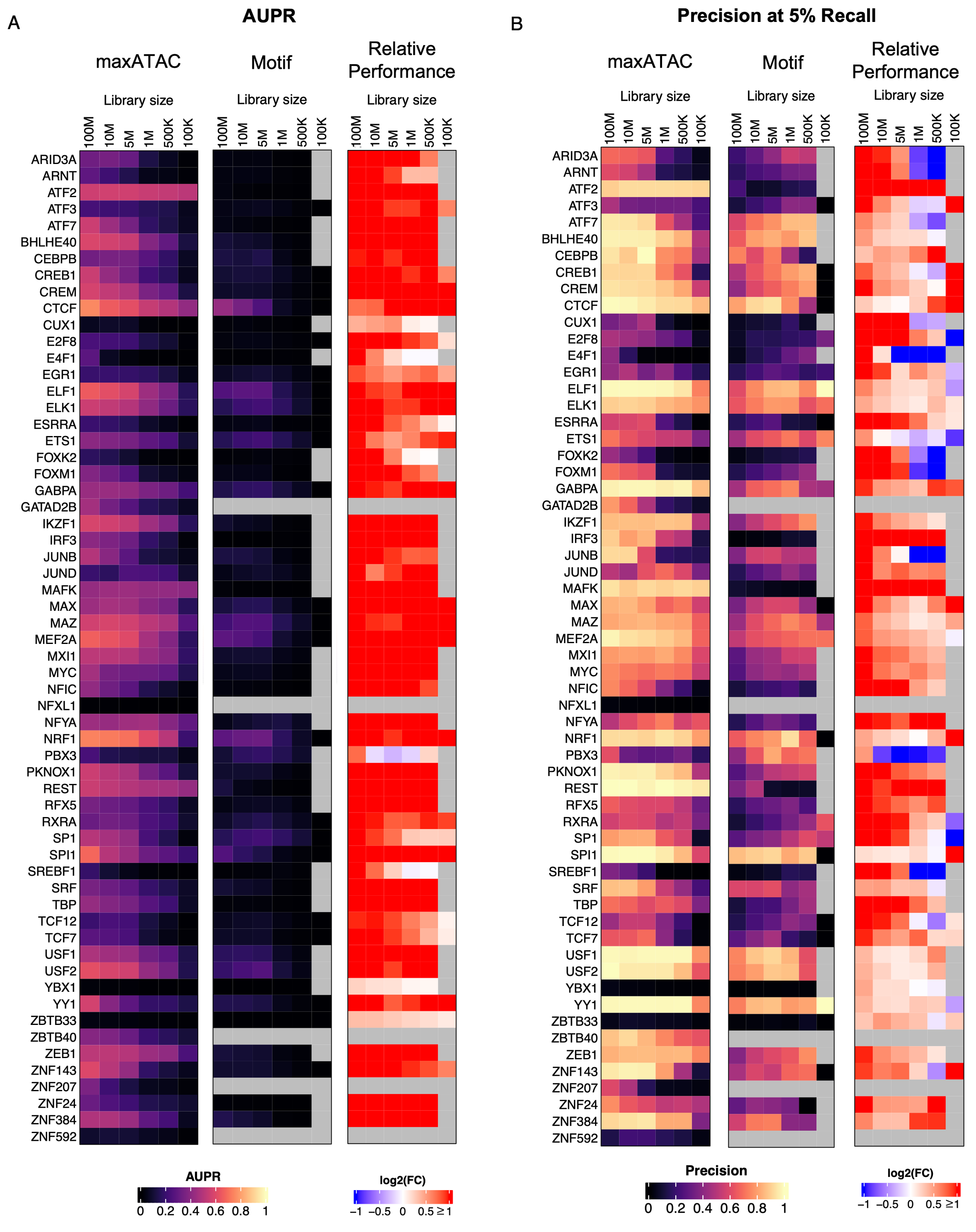

Supplement: S7 Fig — scATAC-seq libraries for GM12878 were down-sampled from 100M to 100k fragments, and test (A) AUPR and (B) precision at 5% recall performances evaluated. Log2(FC) indicates maxATAC performance relative to TF motif scanning for 60 TFs. Gray boxes indicate either (1) no known motif (e.g., GATAD2B, NFXL1, ZBTB40, ZNF207, ZNF592) or (2) no motif predictions for accessible chromatin regions detected at the given library size. (TIF) [file pcbi.1010863.s012.tif]

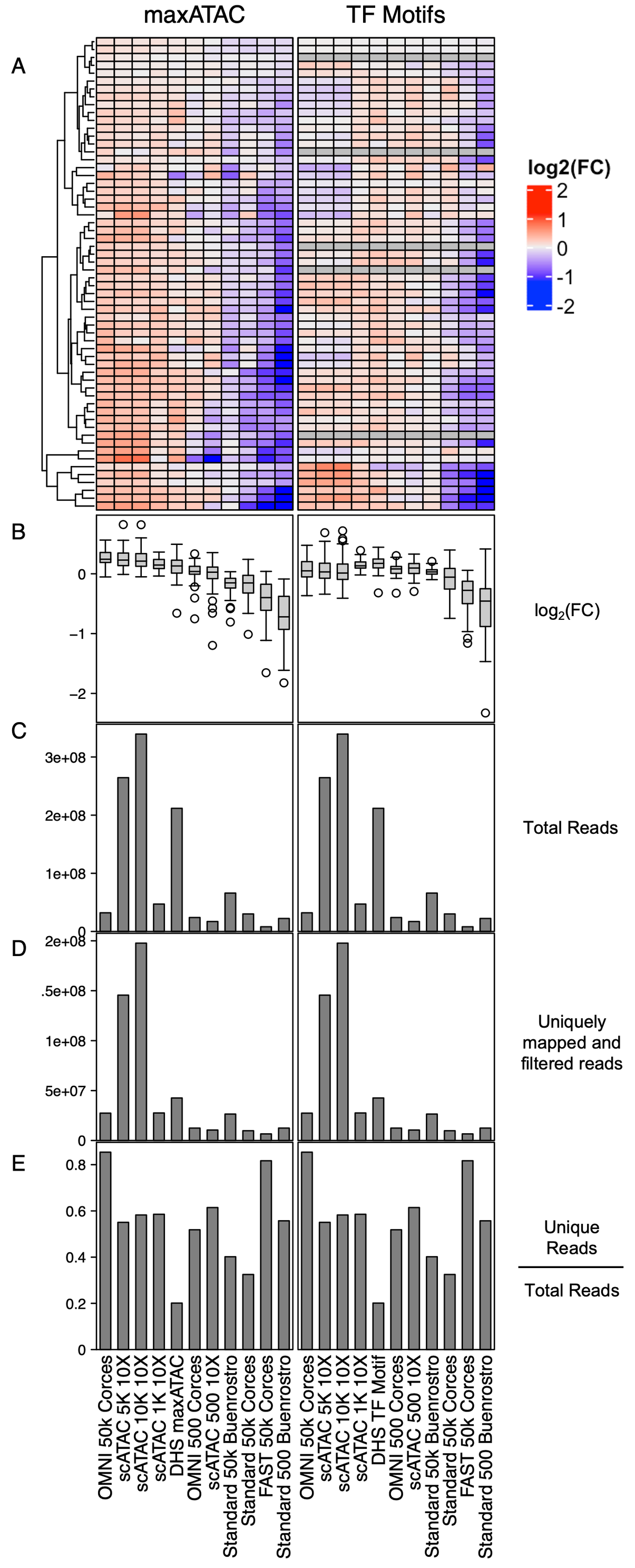

Supplement: S8 Fig — (A) Log2(AUPR:AUPRmean per TF and TFBS method) normalized across ATAC-seq protocols per TF, for maxATAC and TF motif scanning separately. (B) The distribution of log2(AUPR:AUPRmean per TF and TFBS method). (C) Total reads across biological replicates (when available) for each experimental protocol. (D) Total reads that uniquely map across biological replicates. (E) Mean proportion of uniquely mapping reads across biological replicates per protocol. (TIF) [file pcbi.1010863.s013.tif]

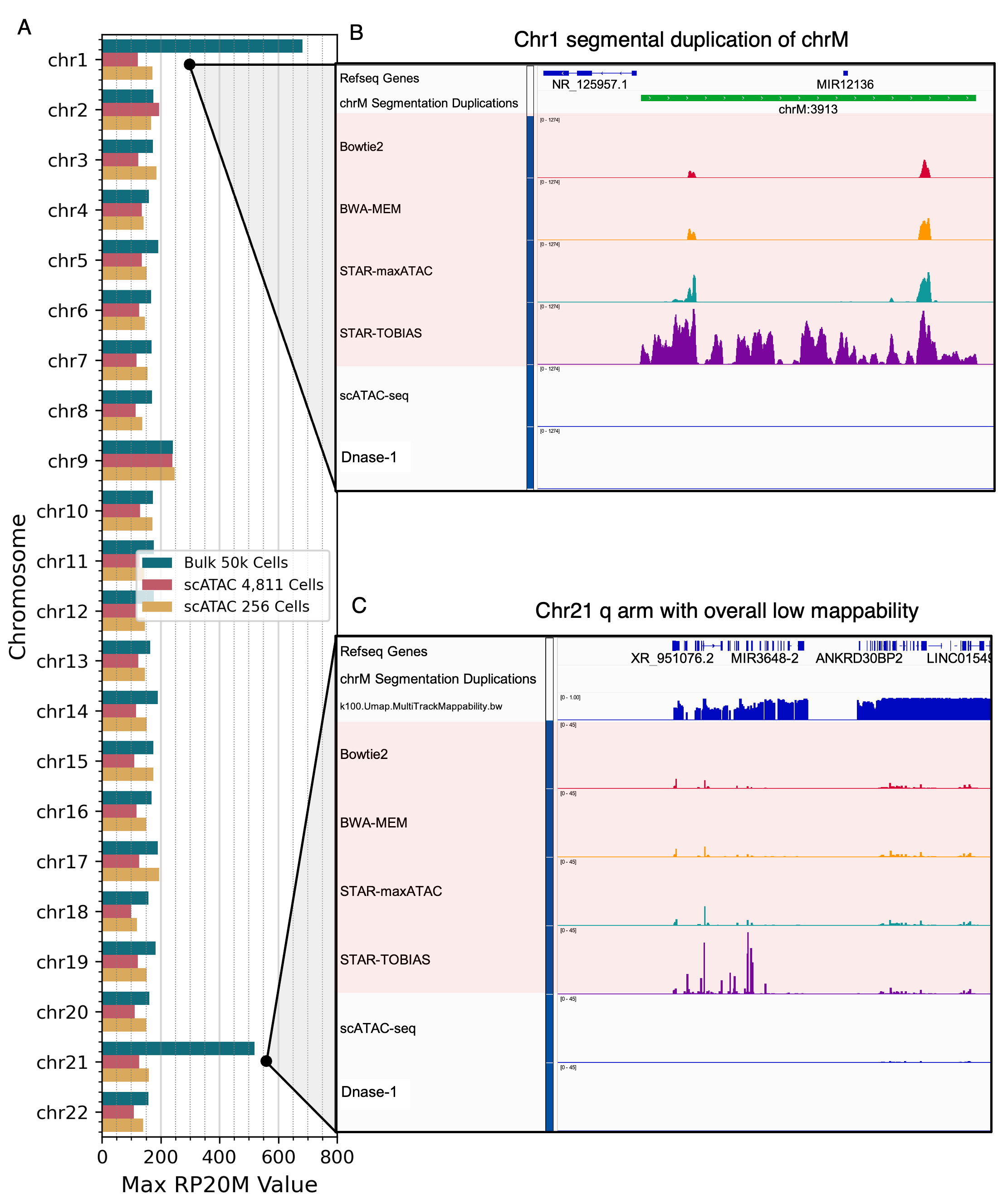

Supplement: S9 Fig — (A) The maximum number of Tn5 cut site counts per autosomal chromosome for bulk OMNI-ATAC-seq (blue), 10x scATAC-seq for 500 cells (yellow) and 5,000 cells (red) in GM12878. (B) IGV screenshot of a mitochondrial chromosome (chrM) segmentation duplication locus (green bar). While chromatin accessibility measurement by DNase-seq and scATAC-seq have no signal in this region, this is a region of extreme signal for OMNI-ATAC-seq, regardless of alignment methods. Pink tracks indicate OMNI-ATAC-seq mapped with multiple alignment methods (Methods). (C) IGV screenshot of the largely unmappable q-arm of chr21 and the relatively high signal caused by low mappability. Again, DNase-seq and scATAC-seq have no signal in this region, while OMNI-ATAC-seq has extreme signal, regardless of alignment strategy. These observations motivated our extended blacklist (Methods). (TIF) [file pcbi.1010863.s014.tif]

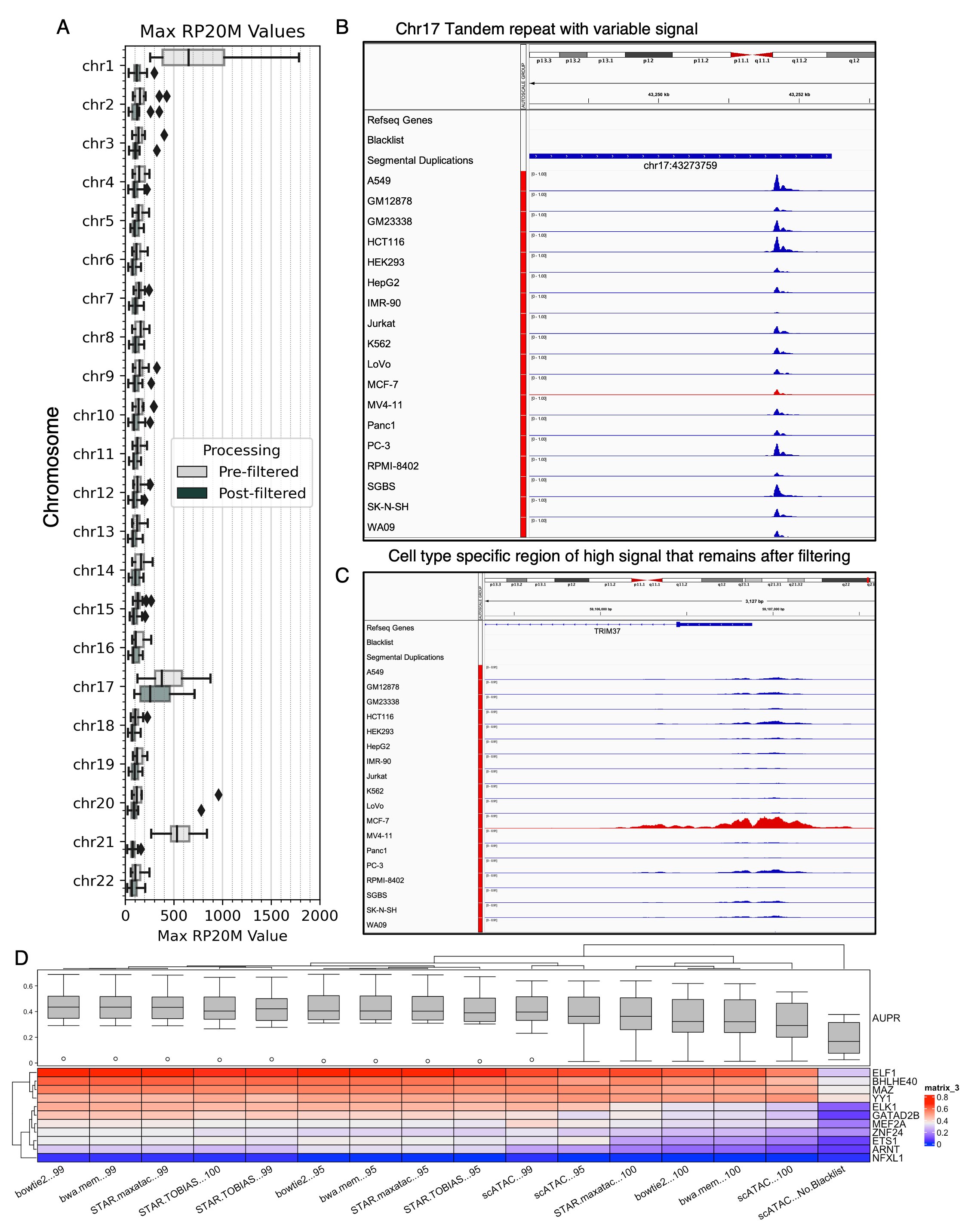

Supplement: S10 Fig — (A) Max RP20M value per autosomal chromosome before and after filtering extended blacklist regions. (B) An example tandem repeat region with variable signal found on Chr17. (C) The TRIM37 locus on Chr17 exhibits extreme, biologically relevant signal in the breast cancer cell line MCF-7 (red track). (D) Test AUPR in GM12878 for different alignment and normalization strategies (Methods) for bulk ATAC-seq or scATAC-seq (pseudobulk of 5k GM12878 cells). PCR duplicates and Tn5 cut sites that mapped to the extended blacklist (Methods) were removed prior to normalization. Test data is bulk ATAC-seq unless described as "scATAC". "95", "99", and "100" correspond to minmax normalization to the 95th, 99th or 100th-percentile highest ATAC-seq signal. "100" therefore corresponds to standard minmax normalization to the absolute max; this strategy was not robust to outlying ATAC-seq signal and therefore performs poorly when applied to different ATAC-seq alignment strategies or scATAC-seq. The far-right column represents performance on scATACseq data using (1) standard min-max normalization and (2) without applying the extended blacklist; this strategy has the worst performance generalizability to scATAC-seq. (TIF) [file pcbi.1010863.s015.tif]

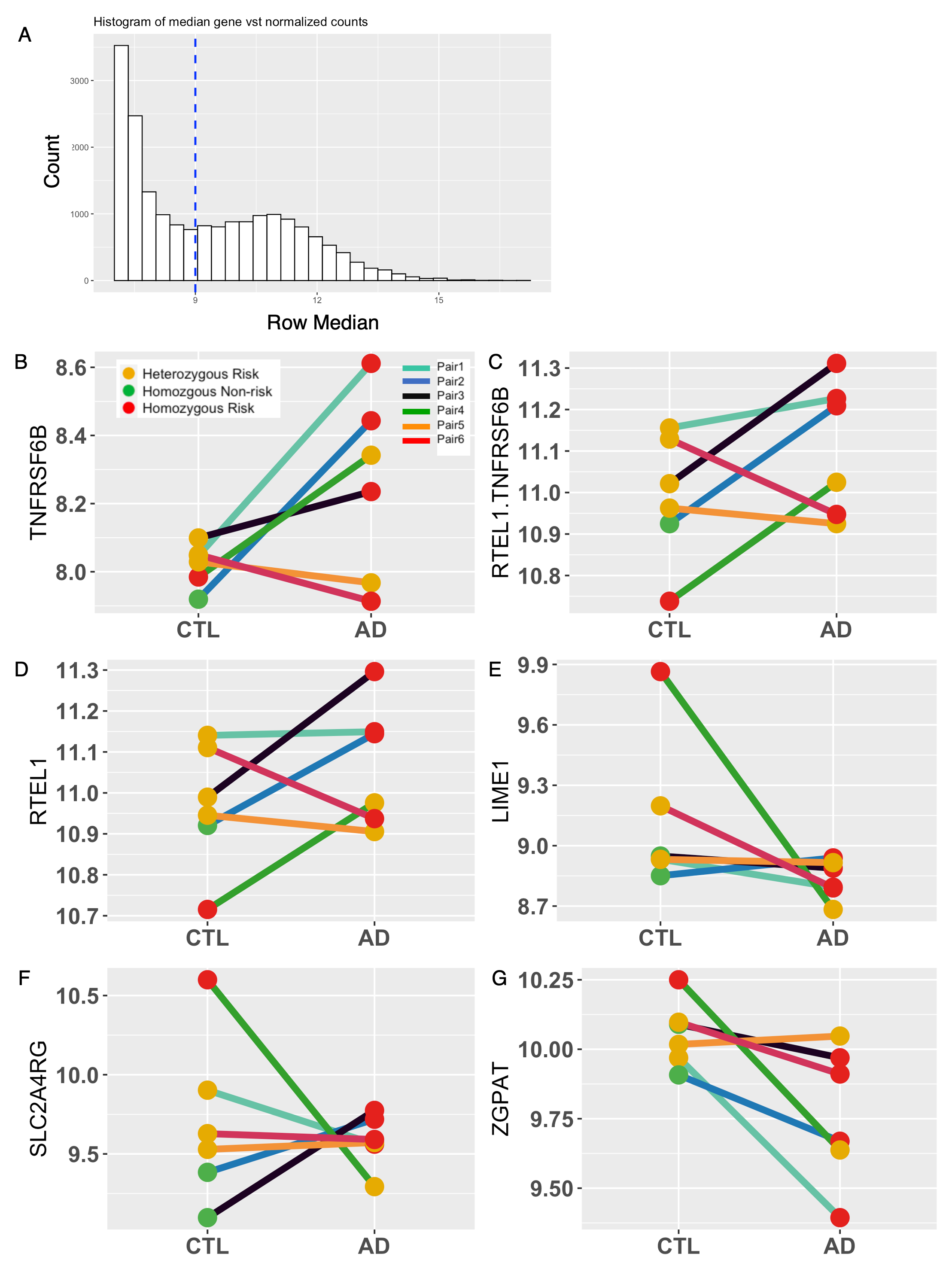

Supplement: S11 Fig — (A) Median expression for each gene (DESeq2 VST-normalized counts) in the activated T cells RNA-seq dataset (6 AD patients and 6 age-matched controls). Blue dotted line indicates the nominal gene expression cutoff applied. Paired line plots showing the difference in gene expression between AD patients and their age-matched controls for (B) TNFRSF6B, (C) RTEL1-TNFRSF6B, (D) RTEL1, (E) LIME1, (F) SLC2A4RG, and (G) ZGPAT. Each point is colored according to whether the donor was homozygous risk (red), heterozygous risk (yellow), and homozygous non-risk (green). Each line is colored according to the donor pair. (TIF) [file pcbi.1010863.s016.tif]

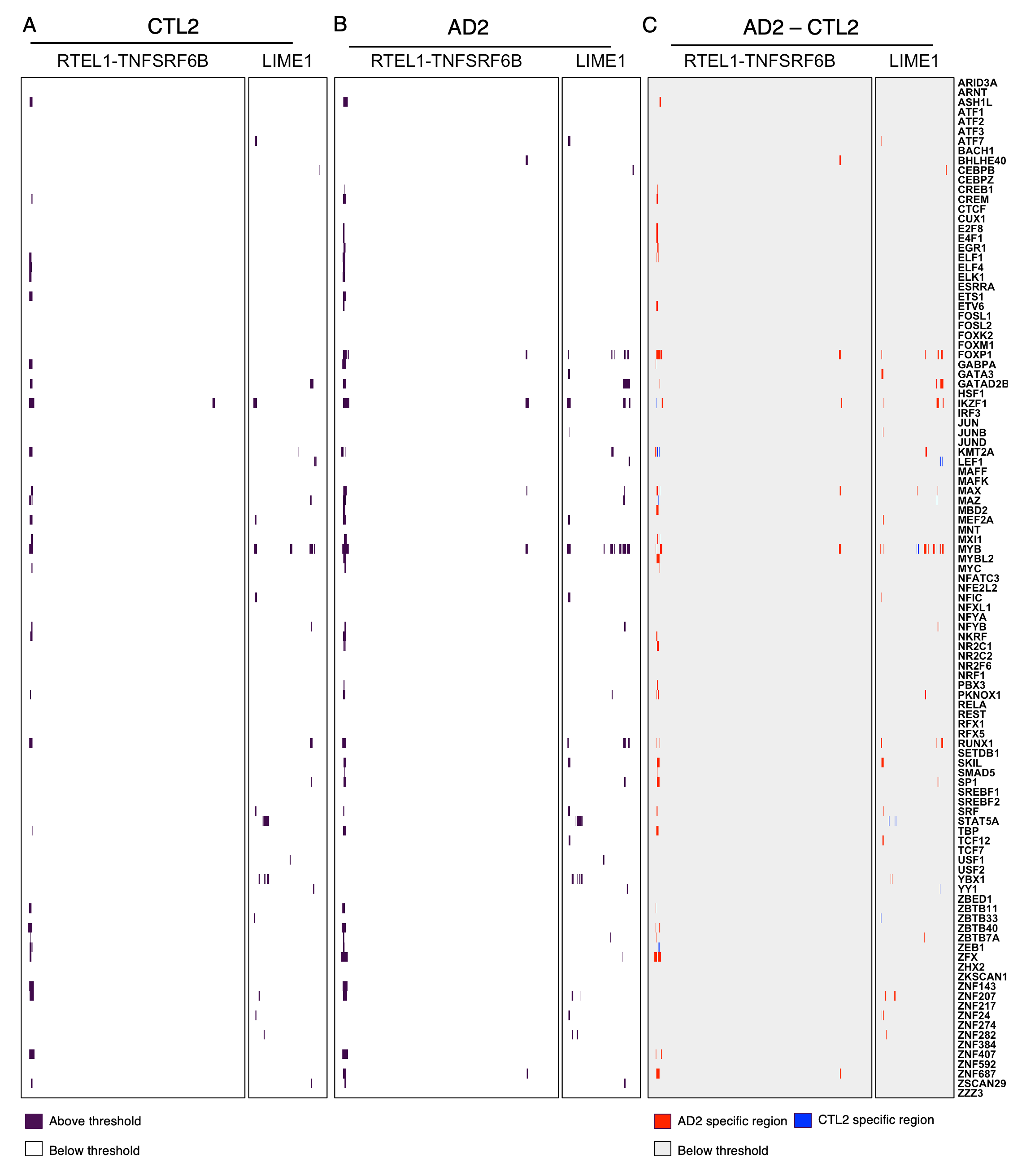

Supplement: S12 Fig — Heatmaps show TFBS predictions (32bp width) for (A) CTL2 and (B) AD2, using a score cutoff that maximizes the average F1-score across validation cell types. (C) Differential TFBS between AD2 and CTL2, where red indicates AD2-specific prediction, blue indicates CTL2-specific prediction and grey denotes no difference. (TIF) [file pcbi.1010863.s017.tif]
