# Supplementary material for: maxATAC: Genome-scale transcription-factor binding prediction from ATAC-seq with deep neural networks
Source: PLoS Comput Biol. 2023 Jan 31;19(1):e1010863. doi: 10.1371/journal.pcbi.1010863 (PMC9917285; doi:10.1371/journal.pcbi.1010863)
Supplement: Attachment — Submitted filename: 221118_maxATAC_responseToReviewers.pdf [file pcbi.1010863.s018.pdf]

Cincinnati Children's
changing the outcome together

Dr. Teresa M. Prztycka
Academic Editor
PLoS Computational Biology

Dr. William Noble
Section Editor
PLoS Computational Biology

November 18, 2022

Dear Dr. Przytycka and Dr. Noble,

Thank you and the reviewers for the constructive feedback on our manuscript. As a result of the review process, our revised manuscript benefited from several new analyses, including (1) additional benchmarking comparisons of our TF binding prediction method, maxATAC, to a state-of-the-art deep learning comparator Leopard and the "footprinting" method TOBIAS and (2) interpretation analyses that uncovered canonical and noncanonical TF motifs learned by the maxATAC models. We also improved (1) communication of the methods through additional figures and text edits and (2) context via citation and discussion of two relevant studies suggested by Reviewer 1.

Below we respond to all reviewer comments in detail.

Thank you, again, to you and reviewers for the reviews. We appreciate your contributions to this manuscript.

Sincerely,

Emily R. Miraldi

Emily Miraldi, PhD
Assistant Professor
Divisions of Immunobiology and Biomedical Informatics
Cincinnati Children's Hospital

Cincinnati Children's Hospital Medical Center
3333 Burnet Avenue, MLC 7038, Cincinnati, OH 45229-3039 I 513-636-0281I Fax 513- 636-5355 www.cincinnatichildrens.org

__**Comments to the Authors & Response to Reviewers:**__

Reviewer #1 Comments:

The authors have presented a computational framework (maxATAC) based on deep convolutional neural networks for predicting transcription factor (TF) binding from ATAC-seq profiles. Authors demonstrated TF binding side prediction from ATAC-seq in new cell types. For benchmarking, authors curated an extensive dataset of existing cell-type specific ChIP-seq and ATAC-seq datasets. Data were manually verified using annotations of each experiment. Their models performed well on both ATAC-seq and scATAC-seq. Quality-controlled, processed datasets are also available to download. Overall, it is a well-written manuscript with an apt description of the method.

The deep learning approach used in this work is not considered as novel (e.g. Tianqi Yang et al, Bioinformatics 2022, Laiyi Fu et al, Science Advances 2020). While this work is unique on capability of trans-cell TFBS predictions and providing community access for quality-controlled, processed, ready-to-use curated large dataset to advance gene regulatory network research on ATAC-seq data

Author Response

- Thank you for bringing these references to our attention. We now highlight them in the text and amend claims of maxATAC novelty accordingly.
- The method **TAMC** (Yang et al. (2022) *PLoS Comp. Bio.*), is a hybrid foot-printing and deep learning method. Similar to other foot-printing methods (e.g., TOBIAS, HINT-ATAC), TAMC relies on a library of known TF motifs, and its TFBS predictions are limited to binding sites centered on those motifs. This is in contrast to deep learning methods like maxATAC that learn TF motifs de novo (see response to **Reviewer 3 Comments 1-2** for novel TF motifs learned by maxATAC). In TAMC, a deep neural network is used to process ATAC-seq signal, to improve footprint (and TFBS) prediction.
- The method **scFAN** (Fu et al. (2020) *Sci. Advances*) is more similar to maxATAC. Like maxATAC, scFAN predicts TFBS from ATAC-seq signal and (unlike maxATAC) uses a mappability track. They use ATAC-seq and TF ChIP-seq data from three cell lines: K562 (60 TFs), GM12878 (33 TFs) and H1ESC (31 TFs). There is one multitask model per cell line (each trained independently). The goal of the method is to predict TF activities in individual scATAC-seq cells, using the bulk-trained deep learning models. In their manuscript, the scFAN authors test trans-cell type prediction, using the 17 TFs in common among their three training cell lines (their Fig. S3). Inspired by **Reviewer 2 Comment 1**, we attempted to directly compare trans-cell type prediction between the maxATAC and scFAN models, but we ran into issues (detailed in response to **Reviewer 2 Comment 1**).

Reviewer #2 Comments:

This manuscript curated a good quality dataset for ATAC-seq and TF ChIP-seq, and present a method named maxATAC for prediction of TF binding sites. TF binding site prediction is an important problem in gene regulatory analysis, and has broad application in many fields. The curated dataset will be useful for future method developments. The manuscript writing is clear and easy to follow. However, I have one concern about the validation of the method.

R2 Comment 1. *There are several methods available for TF binding site prediction from chromatin accessibility data, for example methods presented in the ENCODE-DREAM challenge. Using different data as input and comparing AUPR is not fair. The reason authors did not compare maxATAC with those methods is the difference of input data. I agree that there are some differences between ATAC and DNase. However, in most cases, these two data are highly correlated. Authors can train those methods on the curated data they collected and perform direct comparison with those methods.*

Author Response

- We agree with the reviewer that **Fig. 3F** is an imperfect comparison, due to differences in training and test data and gold standard ChIP-seq. The purpose of the comparison is to show that maxATAC performance on ATAC-seq is roughly, in-the-ballpark of state-of-the-art methods for TFBS from DNase-seq.
    - Although we retain **Fig. 3F** in the main text, we further highlight the limitations of the comparison, to aid reader interpretation.
    - More importantly, we added an additional analysis comparing maxATAC to Leopard, the state-of-the-art successor to ENCODE-DREAM Challenge methods (new **Fig. 3G and S4G**). On average, the maxATAC models outperform Leopard models on ATAC-seq, for the 29 TF-cell type combinations available for test performance.
- Although we compared to Leopard, we would have liked to include additional comparisons to other trans-cell type, deep-learning TFBS methods: Factornet, DeepGRN and scFAN. (Thanks to Reviewer 1, we became aware of scFAN, a deep learning method for TFBS prediction from ATAC-seq, and so an ideal comparator.) Below we detail some of the technical issues that hindered our efforts and rendered additional method comparisons beyond the scope of our study:
    - We attempted to benchmark against scFAN, DeepGRN, and FactorNet for this review, but were unable to run these methods.
        - Factornet is no longer supported and cannot be installed in newer python3 code, because it is based in python2.
        - DeepGRN and scFAN cannot be installed as provided from their published code on GitHub (see https://github.com/sperfu/scFAN/issues/4 and https://github.com/jianlin-cheng/DeepGRN/issues/1). We alerted authors about codebase usability issues.
        - In addition, there were missing details regarding how DNAse-seq or ATAC-seq signal inputs were processed, making it impossible to replicate inputs.
    - Direct input of maxATAC-curated data to the existing TFBS methods is not trivial, because our ATAC-seq data is aligned to the hg38 genome, and all methods using the ENCODE-DREAM Challenge are hard-coded for hg19, including Leopard. This is an issue because these methods rely on accurate chromosome sizes, blacklists, mappability tracks, and genome feature tracks for input.
    - We would like to highlight that maxATAC codebase usability was a central focus of our study. Our codebase (https://github.com/MiraldiLab/maxATAC) includes detailed walkthroughs, issue tracking, ready-to-go data inputs/outputs and working examples. maxATAC is also capable of flexibly incorporating different reference genomes. Below is a table that lists some of the difficulties of implementing current state-of-the-art methods compared to maxATAC:

Method	Hardcoded for hg19	Output Format	Currently Supported	Can be installed?	Scripts to reproduce inputs	hg19-specific feature tracks (i.e., mappability; average signal)
TOBIAS	N	BED	Y	Y	Y	N
DeepGRN	Y	Bigwig	N	N	N	Y
scFAN (bulk)	Y	Numpy array	Y	Y (with modifications)	N	Y
FactorNet	Y	Bigwig	N	Y (with modifications)	N	Y
Leopard	Y	Numpy array	Y	Y	Y	Y
maxATAC	N	BED + Bigwig	Y	Y	Y	N

- Comparison of neural network architectures (training state-of-the-art DNase-seq methods on our ATAC-seq benchmark) is nontrivial, beyond the scope of the current study and the subject of future work. Training existing models on the maxATAC benchmark (even a subset of the 74 TF benchmarks, train-test cell type splits) requires substantial resources (both compute and personnel time). For new **Figs. 3E, S4G**, we used the DNase-trained Leopard models to predict TFBS from ATAC-seq, finding that Leopard performance was almost on par with maxATAC's.
- We rigorously demonstrate that maxATAC's performance is more than a function of network architecture. Training strategies for our sparse data (**Fig. S2A**) and ATAC-specific normalization strategies (**Fig. S9, S10**) were key to maxATAC performance. Furthermore, **Fig. 5** analyses, testing of ATAC-trained maxATAC models on DNAse-seq (and even other ATAC-seq protocols), rigorously show that data-type-specific training is needed for optimal performance on that datatype. Collectively, our results (including benchmarking across methods and chromatin accessibility data types **Fig. 3C-F**, **4-6**, **S4A-G**) provide compelling evidence for application of maxATAC to ATAC-seq data. We anticipate that maxATAC performance will serve as a baseline and that our well-organized codebase (GitHub) and benchmark (Zenodo) will spur advances by many computational groups, including training of their existing algorithms on the maxATAC benchmark. The performance of DNase-trained Leopard models is promising.
- In addition to testing existing architectures, we are eager to test transformer architectures and top-performing methods, soon to be released from the 2022 DREAM Challenge "Predicting Gene Expression Using Millions of Random Promoter Sequences". These are important future directions and the subjects of on-going work.

R2 Comment 2. *To evaluate the motif scanning, how authors calculate the AUPR is not clear. I assuming they slide the motif matching score to calculate the AUPR. Another way to perform comparison is sliding sum of (or product of) motif matching score and ATAC-seq signal (RPKM, or openness) to calculate AUPR.*

Author Response

- To improve communication of the AUPR calculation, we added a new schematic (**Fig. S3**), to complement text description and equations in **Methods**. In brief, for TF-motif-based prediction, we rank TFBS predictions based on the number of TF motif occurrences in a 200bp region. By this method, MACS2-peak-calling is used to limit motif scanning to accessible chromatin regions, and the quantitative nature of the ATAC-seq signal is not leveraged.
- In the revised manuscript, we additionally benchmark maxATAC relative to a more sophisticated method, TOBIAS, that integrates ATAC-signal intensity (foot-printing) with motif scanning (see new **Fig. S5**) for comparison to TOBIAS:
    - maxATAC outperforms TOBIAS in terms of AUPR and precision at 5% recall (**Fig. S5A,B**).
    - We also compared our motif-scanning approach to TOBIAS (**Fig. S5C,D**): As expected, motif-scanning outperforms TOBIAS in terms of AUPR, but, on average, TOBIAS has higher precision at low recall than motif scanning.

Reviewer #3 Comments:

The Cazares et al. manuscript presents a well-curated benchmark dataset that pairs ATAC-seq and published transcription factor (TF) ChIP-seq in 20 cell types for an unprecedented number of TFs (127 TFs with ChIP-seq data in at least 2 cell types; 74 TFs of them with data in at least 3 cell types). The authors generated their own OMNI-ATAC-seq for some cell lines to assemble the resource. In addition to the dataset, the paper proposes a deep learning model that predicts trans-cell-type TF occupancy based on a dilated CNN model. The maxATAC model was carefully evaluated using both bulk and single-cell ATAC-seq data on various cell types and TFs in a held-out chromosome and held-out cell type manner and was shown to reach comparable (though perhaps slightly weaker) performance to several current state-of-the-art methods for cross-cell-type TF occupancy prediction developed for DNase-seq.

The maxATAC approach also had superior performance compared to traditional motif scanning or ChIP-seq signal averaging across training cell types for most TFs.

Overall, this manuscript is well-written and results are nicely presented, with the limitations of the study helpfully described in the Discussion. While there is limited technical or conceptual novelty relative to previous neural network models that tackle the same or related problems, the benchmark dataset establishes a useful resource, and the maxATAC performance results lay down a useful baseline for future deep learning methods. More exploration of the central question of cross-cell-type generalizability of TF occupancy prediction, wider method comparison, and better interpretation of the trained models would strengthen the paper.

R3 Major points:

1. *The key question for the paper is the extent of generalizability of TF binding models to new cell types, where potentially new co-factors or members of the TF complex may be expressed and alter the sequence recognition code. The authors present various analyses to explain the variability in maxATAC's performance over TFs, but the issue of the cell-type-specificity of the underlying sequence signal is not fully developed. For example, in Figure S5, the authors show the maxATAC auPR relative to training ChIP-seq signal auPR (as a log odds ratio) vs Jaccard distance over the training ChIP-seq samples. This tells us that when a factor binds nearly the same sites in all cell types (e.g. CTCF), it is hard to outperform training ChIP-seq signal, whereas the model can outperform this baseline when there are cell-type-specific sites. However, it absolute terms, maxATAC has the highest auPR on CTCF test data out of all factors, because it also has a highly conserved binding motif across cell types. So it would be helpful to address how the model performs on unseen cell types for TF with cell-type-specific binding motifs as compared to highly conserved binding motifs.*

2. *As a related issue, a model interpretation analysis, e.g. using feature attribution to identify cell-type-specific vs. conserved motifs, might also help investigating the issue of generalizability. In particular, is the model actually learning co-factor binding signals in addition to the TF motif? Can the authors show that the model is learning multiple modes of binding for TFs with cell-type-specific binding motifs/patterns?*

Author Response

We address **Comments 1 & 2** together. We are very interested in questions of model interpretability and cell-type-specificity, and this is the focus of a follow-up manuscript. However, inspired by your suggestions, we highlight two examples in the main text, see new section: "**maxATAC model performance gains correlate with cell-type specificity**" and new **Fig. S6**:

"We used model interpretation techniques to determine context-specific DNA sequence patterns (TF motifs) learned by the maxATAC models. We highlight two examples: GATA3 and CREM, which exhibit high (GATA3) and moderate (CREM) cell-type-specificity (**Fig. S5C**). To identify the DNA sequences (TF motifs) driving maxATAC TFBS prediction, we applied the TF-MoDISco method (Shrikumar et al. (2018) *arXiv*) to the final maxATAC models (trained using all available cell types). These analyses uncovered cell-type-shared and cell-type-unique motifs, which correlated with test performance:

- **GATA3.** Across the four training cell types, we identified three distinct motifs that correspond with known GATA motifs: (1) monomer, (2) dimer with 3bp gap and (3) dimer with 4bp gap (**Fig. S6A**). The 3bp-gapped dimer motif was not detected in A-549. Interestingly, test AUPR for A-549 (.43) was lower than AUPRs for the other cell types (.51 (SK-N-SH), .54 (MCF-7) and .56 (Jurkat)), suggesting that a model constructed using SK-N-SH, MCF-7 and Jurkat might have lower test performance on A-549 due to recognition of a motif (the 3bp-gapped dimer motif) that was not

bound by GATA3 in A-549. This suggests the existence of cell-type specific co-factors or post-translational modifications for GATA3 that are not present in A-549 to mediate DNA binding to the 3bp-gapped dimer motif.
- **CREM**. Across the three training cell types, we identified three motifs: (1) the classic AP-1 7-base motif ("AP-1 short"), (2) the classic AP-1 8-base motif ("AP-1 long"), which has an extra base pair between half-sites, and (3) "AP-1 (methyl)", an AP-1 motif with the extra base pair between half sites, but with "TG" replaced by a likely methylated "CG", as frequently observed in *in vitro* methylated DNA binding data (Yin et al. (2017) *Science*) (**Fig. S6B**). Although the three motifs are identified across each of the cell types, their frequency varies substantially. The frequency of the AP-1 short and AP-1 long motifs is roughly halved in HepG2, relative to GM12878 and K562, suggesting that the maxATAC-learned TF motifs don't explain CREM binding in HepG2 as well as GM12878 and K562. In line with this reasoning, HepG2 test AUPR (.53) is the lowest, relative to GM12878 (.58) and K562 (.64).

Collectively, these results demonstrate that the maxATAC models are learning both canonical and noncanonical TF motifs and suggest (1) that cell-type-specific TF binding patterns not observed in training cell types, limit test performance in cell types with that pattern (GATA3 example), and (2) differences in the frequencies of TF motifs recognized in training versus test cell types might impact generalizability (CREM example)."

As noted above, maxATAC model interpretation is the subject of a future manuscript. As an important complement to the model interpretation analyses, we will also continue to directly assess questions of maxATAC model generalizability by directly testing models in new cell types (e.g., as we did in primary T cells, **Fig. 6**).

3A. The major method comparison was made between maxATAC and two baseline methods (motif-scanning and ChIP-seq average signal), but since there are also some published ATAC-seq based TF "footprinting" approaches such as HINT-ATAC and TOBIAS, i.e. methods that try to model the ATAC-seq signal to better local the potential TF binding site, it would be interesting to see a comparison with them.

Author Response
- Thank you for this suggestion. We now include a comparison to TOBIAS in **Fig. S5**. As summarized in response to **Reviewer 2 Comment 2**: maxATAC outperforms TOBIAS in terms of both AUPR and precision at 5% recall (**Fig. SA-B**).

3B. It would also be interesting to understand whether the maxATAC model is learning a "footprinting" method (relatively protected region within a peak) or simply finding peak summits.

Author Response

We used deep neural network activation maximization techniques (Simonyan et al. (2013) *arXiv*; doi: https://arxiv.org/abs/1312.6034v2) to determine whether the maxATAC models learn (1) "footprints" from the ATAC-seq signal input or (2) simply high-signal ATAC-seq peak regions / summits from the training data. Specifically, we identified the signal pattern in the ATAC-seq input channel that maximized the model score for positive TFBS prediction, while keeping the DNA sequence input channels unchanged during the gradient ascent updating process. Here, we first provide an example using the final ATF2 model and a positive TFBS (ChIP peak) on test chromosome 1 from training cell type GM12878. The activation maximization process yielded a "footprint-like" ATAC-seq signal pattern centered on the ChIP-seq (**Fig. R2A**), after 5 steps of gradient ascent updates in the ATAC-seq input channel (learning rate of 5.0). Aggregating optimal ATAC signal for all positive (ChIP-seq peak centered) versus negative (ATAC-seq-centered but no ChIP peak) binding examples (test chromosomes, GM12878) for the ATF2 model

shows that the model has learned to recognize a relatively protected region of approximately -25 to 25 bps where the cleavage pattern will take place, while negative examples show highest signal intensity almost right in the middle of the receptive field (**Fig. R2B**). These observations show that the maxATAC models can learn co-localization between the ATAC-seq footprints and highly probable binding regions from the DNA sequences.

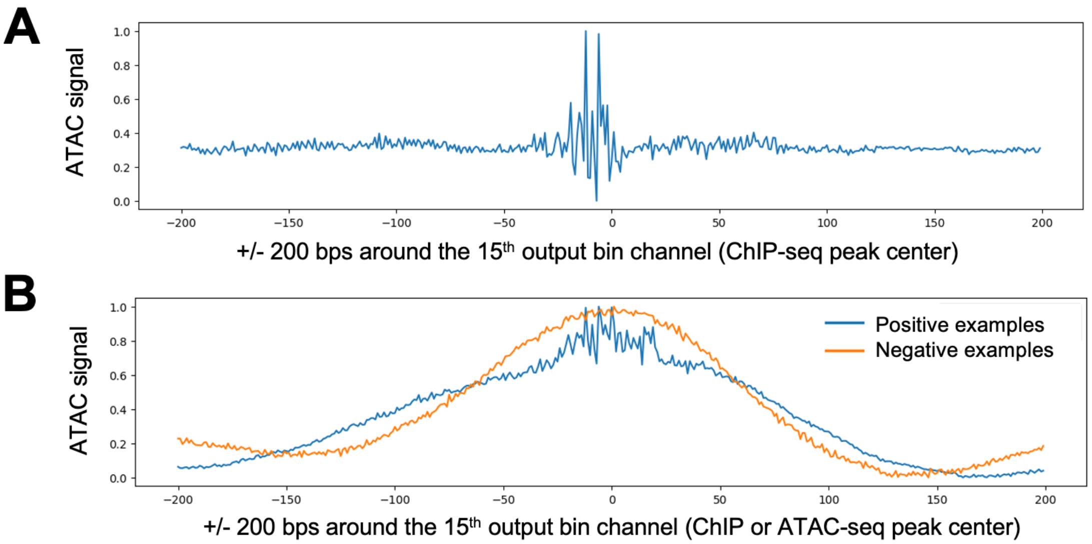

Fig. R2. Identifying ATAC signal pattern that maximizes positive TFBS prediction. (A) ATAC signal optimized for positive ATF2 TFBS prediction using DNA sequence centered on a ChIP-seq peak. Signal is min-max normalized across the 1024bp input window and centered +/- 200bps around the 15th output bin (corresponding to ChIP peak center). **(B)** Optimal ATAC signal for positive ATF2 TFBS prediction, averaged across (1) positive TFBS examples (ChIP peak centered) or (2) negative TFBS examples (ATAC-peak centered but no ChIP support). Signal is normalized and centered as in (A).

Comment 4

It would also be interesting to compare the deep learning method to "shallow learning", e.g. a kernel method like gkm-SVM combined with a simple kernel on ATAC-seq signal.

Author Response

- This interesting comparison was explored by two previous publications:
  - DeepSea (Zhou and Troyanskaya (2015) *Nature Methods*; doi:10.1038/nmeth.3547): Fig. S2 from the DeepSea manuscript compares TFBS prediction between the DeepSea CNN and gkm-SVMs (690 TF binding profiles, 160 TFs), showing superior performance for the CNN.
  - Zeng et al. (2016) *Bioinformatics* (doi:10.1093/bioinformatics/btw255), which compared several CNN architectures (including DeepBind) to gkm-SVMs. They provide guidelines about how to design CNN architectures and benchmarks. Provided these best-practices are followed, CNNs outperform simpler gkm-SVM models.
- Given these previous studies, we focus new comparisons in the revised manuscript to (1) footprinting methods TOBIAS and (2) tests involving existing deep-learning methods for DNase-seq (**see response to Reviewer 2, comment 2**).

Comment 5

There are also some improvements that could be made to make the presentation of this paper clearer. Various model and training details (explicit explanation of the output of the model, ground truth used for training, details of the encoding and 32bp resolution) are not clear until the methods, and some details are obscure even there? It would be helpful to include a figure and overview of the model in the main text. The training set-up might be described in the ENCODE-DREAM challenge, but a self-contained presentation would be helpful here. Also, the wording "a suite of models" and "a collections of models" is

a bit misleading – it is not true that there is collection of models with different structures, as is finally clarified in the model description section, but rather there are models for different TFs (all with the same architecture).

Author Response

- Thank you for the suggestions. We have made the suggested improvements:
    - We include an overview figure of the model in the main text (new **Fig. 2**).
    - We more clearly point to relevant details (training, ground truth) in **Methods** and main text.
    - We more clearly describe what we mean by "suite" or "collection" of models.

Minor points

- Fig S1A: should have a consistent use of cell-line-specific and cell-type-specific in the figure caption
- Line 198: should be sequence-based

Author Response

- Now fixed. Thank you for catching these mistakes.
